# *Trichoderma viride* Laccase Plays a Crucial Role in Defense Mechanism against Antagonistic Organisms

**DOI:** 10.3389/fmicb.2016.00741

**Published:** 2016-05-17

**Authors:** Lakshmanan Divya, C. Sadasivan

**Affiliations:** Department of Biotechnology and Microbiology, Kannur UniversityKannur, India

**Keywords:** antifungal, defense mechanism, inter-specific interaction, laccase, *Trichoderma* sp.

## Abstract

Fungal laccases are involved in a variety of physiological functions such as delignification, morphogenesis, and parasitism. In addition to these functions, we suggest that fungal laccases are involved in defense mechanisms. When the laccase secreting *Trichoderma viride* was grown in the presence of a range of microorganisms including bacteria and fungi, laccase secretion was enhanced in response to antagonistic organisms alone. In addition, growth of antagonistic microbes was restricted by the secreting fungi. Besides, our study for the first time shows the inability of the secreting fungi (*T. viride*) to compete with antagonistic organism when laccase activity is inhibited, further emphasizing its involvement in rendering a survival advantage to the secreting organism. When laccase inhibitor was added to the media, the zone of inhibition exerted by the antagonist organism was more pronounced and consequently growth of *T. viride* was significantly restricted. Based on these observations we accentuate that, laccase plays an important role in defense mechanism and provides endurance to the organism when encountered with an antagonistic organism in its surrounding.

## Introduction

Laccases (E.C.1.10.3.2) are oxidoreductases that contain copper ions at the catalytic center (Kiiskinen et al., 2002) and are one of the few microbial enzymes employed in number of industrial applications (Abadulla et al., 2000; Cuoto and Herrera, 2006; Kidwai et al., 2012; Sole et al., 2012; Divya et al., 2013). Fungal laccases are unique in that they exhibit low substrate specificity and strong oxidative abilities and are involved in a variety of physiological functions such as delignification, morphogenesis, and parasitism (Worrall et al., 1986; Williamson, 1997; Missall et al., 2005; Camarero et al., 2007). In addition to these functions, our study suggests that fungal laccases are involved in conferring the secreting organism a resistance to antagonistic microorganisms.

Besides directly oxidizing a variety of phenolic compounds, laccases catalyze the indirect oxidation of chemicals that are not phenols or amines in the presence of a redox mediator or Laccase-mediator system (LMS), which can be of natural or synthetic origin (Eggert et al., 1998). The combination of the laccase with low molecular weight mediators not only lead to higher rates and yields in the transformation of laccase substrates but also add new oxidative reactions to the laccase repertory toward substrates in which the enzyme alone had no or only marginal activity. Thus, LMS enlarges substrate range being able to oxidize compounds with redox potential (E°) higher than that of laccase.

Most fungi will come across competitive or antagonistic organisms in their natural population and communities. Though these interactions may not produce noticeable morphological response between the intermingling fungi, they can form mutual inhibition zones (Rayner and Boddy, 1988; Cooke and Whipps, 1993). These fungal interactions can be seen both in culture as well as in their natural environment (Crowe and Olsson, 2001; Wei et al., 2010). Studies shows that fungi involved in such competition often produce secondary metabolites, extracellular phenol-oxidizing enzymes like laccase, and differentiated structures in the zone of interaction (Iakovlev and Stenlid, 2000; Crowe and Olsson, 2001; Zhang et al., 2006; Wei et al., 2010). The present study was undertaken to analyze the probable role of laccase in fungal defense mechanisms.

### Laccase Secretion Was Enhanced When *Trichoderma viride* NFCCI-2745 Was Grown in the Presence of Antagonistic Fungi and Bacteria

The laccase producing *Trichoderma viride* Pers NFCCI-2745, which was isolated from a highly saline and phenolic rich environment (Divya et al., 2014) was used for studying the role of laccase in antagonistic microbial interaction. Identification of the strain was done based on Inter Transcribed Spacer (ITS) sequencing of rDNA of fungal genome from the National Fungal Culture Collection of India (NFCCI), Pune, India and the sequence deposited in GenBank under the GenBank accession ID: KF638399.

**FIGURE 1:**
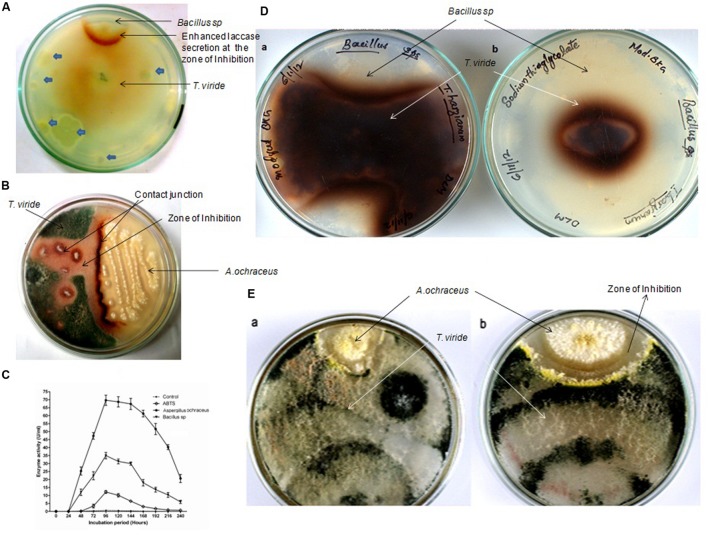
**Representative pictures of the pairing experiments of *Trichoderma viride* with *Bacillus* sp. and *Aspergillus ochraceus.* (A)** Backside of the co-cultured screening plate (1 mM Guaiacol) showing enhanced laccase secretion by *T. viride* in response to the zone of inhibition produced by *Bacillus* sp. Other microorganisms failed to induce any sort of laccase induction (is shown with blue block arrows). **(B)** Plate showing enhanced laccase secretion by *T. viride* response to the antagonistic effects exerted by the *A. ochraceus*. **(C)** Quantitative assay of total laccase activity in the presence of antifungal metabolites. **(D)** Rear view of the Co-Culture of *T. viride –Bacillus* in 3 mM Guaiacol media (a) Control without Laccase inhibitor (b) with Laccase inhibitor. In the control where laccase inhibitor was not added, *T. viride* exhibited normal growth pattern and restrict the growth of *Bacillus* sp., whereas when laccase is inhibited, the growth of *T. viride* was significantly inhibited. Also the pattern of growth exhibited by *T. viride* showed marked difference in the presence and absence of laccase. **(E)** Front view of the Co-Culture of *T. viride –A.ochraceus;* (a) Control without Laccase inhibitor (b) with Laccase inhibitor. In the control where laccase inhibitor was not added, *T. viride* could restrict the growth of *A. ochraceus*. Also *A. ochraceus* failed to exert a prominent inhibition zone. Whereas when laccase was inhibited, the zone of inhibition produced by *A. ochraceus* was remarkably prominent when compared to control.

Top soil along with decomposing leaves and twigs near the canteen premises of the Kannur University Campus, Kerala, India, were sampled for laccase inducing microorganisms. Serially diluted samples were spread plated onto agar plates containing 1 mM guaiacol. Plates were incubated at room temperature for 6 h and then one colony plug of 2 mm diameter were cut out from actively growing edge of *T. viride* NFCCI-2745 and then transferred onto the same screening plate at the center. Only two strains of microbes were found to induce laccase secretion and interestingly both exhibited antifungal properties (**Figure 1**). These two strains were identified as *Bacillus* sp. (**Figure 1A**) and *Aspergillus ochraceus* (**Figure 1B**) based on biochemical and morphological characteristics, respectively. *Bacillus* sp. and *A. ochraceus* were unable to oxidize guaiacol directly but induced laccase secretion of *T. viride* (**Figures 1A,B**). An enhanced laccase secretion was observed around the clear inhibitory zone of *Bacillus* sp. and at the contact junction of *A. ochraceus*. The pattern of induction suggested that the *T. viride* mycelium was reacting to agents diffusing from the antagonistic microbes within the agar medium. The similar form of laccase induction during inter-specific microbial interactions was reported with many fungi belonging to Basidiomycete family as well (Iakovlev and Stenlid, 2000; Crowe and Olsson, 2001; Baldrian, 2004; Qian and Chen, 2012).

In the interaction combinations, laccase activity was high in the contact zone but low or not detectable in other parts of mycelia from 2nd to 4th day in guaiacol (1 mM) supplemented agar medium. After 4 days, laccase activities were distributed more homogeneously over the entire mycelium and became higher in the contact zone compared to initial days of interactions. After 4 days of incubation, *T. viride* started growing above the zone of inhibition formed by the antagonistic organism, restricting the growth of antagonistic microorganism to a great extent, with both forming mutual inhibitory zones. The qualitative assays were confirmed by quantitative assay of total laccase activity (**Figure 1C**). It was found that enzyme activity was induced to almost 50 times in the case of *Bacillus* sp. and almost 100 times with *A. ochraceus*. In the control the laccase activity reached its maximum within 96 h of incubation and slowly declined thereafter. But with the addition of cell free supernatant of the antagonistic culture, the laccase activity reached its maximum within 96 h and remained the same for almost 144 h and then declined slowly. The results of both quantitative and qualitative assays confirmed enhanced laccase secretion as a result of inter-specific fungal interactions.

In contrast to previous studies where *Trichoderma* sp. are reported as inducers, enhancing the laccase production of Basidiomycetes such as *Pleurotus ostreatus, Agaricus bisporus*, *Lentinula edodes, Serpula lacrymans* and *Trametes versicolor* (Score et al., 1997; Savoie et al., 1998; Savoie and Mata, 1999; Hatvani et al., 2002; Baldrian, 2004, 2006; Velázquez-Cedeño et al., 2004; Flores et al., 2009, 2010; Bertrand et al., 2013; Sjaarda et al., 2015), our study for the first time reports enhanced laccase activity in *Trichoderma* sp. in response to other co-cultures. Though many early studies on inter-specific and intra-specific microbial interactions have observed similar induction in laccase secretion in presence of competing microbes (Savoie et al., 1998; Iakovlev and Stenlid, 2000; Crowe and Olsson, 2001; Baldrian, 2004; Velázquez-Cedeño et al., 2004; Snajdr et al., 2011; Qian and Chen, 2012; Sjaarda et al., 2015), its role in conferring a survival advantage to the secreting organism was not probed.

### Is Laccase Secretion in *Trichoderma viride* NFCCI-2745 Necessary for Combating Antagonistic Microbes?

In order to check whether enhanced production of laccase by *T. viride* in the co-culture is necessary for its survival, we decided to conduct a study with laccase inhibitors. Interestingly in the pairing experiments, when laccase inhibitor, sodium thioglycolate (10 mM) was added to the media, we observed a marked difference in the growth pattern of *T. viride* (**Figures 1D,E**). When *T. viride* was paired with *Bacillus* sp. we observed that in the control where laccase inhibitor was not added, *T. viride* exhibited normal growth pattern and restricted the growth of *Bacillus* sp., whereas when laccase is inhibited, the growth of *T. viride* was significantly reduced (**Figure 1D**). The diameter of *T. viride* colony in its pairing with *Bacillus* sp. is shown in **Table 1**. In the pairing experiment with *A. ochraceus*, in the control where laccase inhibitor was not added, *T. viride* could restrict the growth of *A. ochraceus*. Also *A. ochraceus* failed to exert a prominent inhibition zone. Whereas when laccase was inhibited, the zone of inhibition produced by *A. ochraceus* was remarkably prominent (**Figure 1E**). The measurement of the zone of inhibition exerted during this pairing experiment is shown in **Table 1**. These results suggest that the formation of this inhibition zone is influenced by laccase. This result was not influenced by the presence of guaiacol (laccase indicator) in the media. Both the cultures with and without guaiacol exhibited almost the same pattern of interaction. The above results suggest that laccase secretion may be a requisite for *T. viride* to compete with other antagonistic microbes. The results point toward the possibility of the role laccase in conferring defense against antifungal secreting microorganisms. This result supported the previous interpretations, where the changes in laccases production due to interaction of the laccase secreting fungi (Basidiomycetes) and the antagonists (*Trichoderma* sp.) was linked to defense reaction of the secreting fungi which limits the progression of antagonists (Savoie et al., 2001; Velázquez-Cedeño et al., 2007; Sjaarda et al., 2015). In addition, our study for the first time shows the inability of the secreting fungi (*T. viride*) to compete with antagonistic organism when laccase activity is inhibited, further emphasizing its involvement in rendering a survival advantage to the secreting organism.

**Table 1:** Measured parameters of the pairing experiments of *Trichoderma viride* with *Bacillus* sp. and *Aspergillus ochraceus.*

	Control (cm)	With laccase inhibitor (cm)
Diameter of the colony of *T. viride* during pairing with *Bacillus subtilis*	6.76 ± 0.68	2.73 ± 0.25

	**Control (mm)**	**With laccase inhibitor (mm)**

Annular radius of the inhibitory zone in *T. viride* and *A. ocharaceus* pairing	4.5 ± 1.29	7.5 ± 0.58

We also observed that only lyophilized crude cell free supernatant enhanced laccase secretion whereas synthetic antagonistic compounds [Ketoconazole and clotrimazole (50 μl of 150 mg/ml)] as well partially purified antagonistic fraction [chloroform: methanol (7:3)] fraction from culture supernatant of *Bacillus* sp. failed to induce laccase secretion. A previous study by Crowe and Olsson (2001) supports our observations which states that purified antagonistic compound couldn’t induce laccase secretion while the organism (from which the substance was purified) enhanced laccase secretion (Crowe and Olsson, 2001). The authors concluded that it is the calcium or heat shock signaling in response to the effects of bacterial metabolites which induces laccase secretion (Crowe and Olsson, 2001). Though we couldn’t explain the nature of induction of laccase, we believed that laccase may be neutralizing the effects of these toxins, with help of mediators. Some of the earlier studies reports that laccase mediators synthetic as well as natural aids in oxidizing non phenolic substrates by laccase (Thurston, 1994; Eggert et al., 1998). Based on the previous evidences from literatures, we speculate the possibility of certain metabolites secreted by the antagonistic organism in acting as a mediator for laccase, in oxidizing the anti- fungal compound and thus eliminating its antagonistic effect. This may perhaps explain the inability of the purified antagonistic compound to induce laccase secretion. Thus in conclusion laccase may be exerting a significant role in defense mechanism and might be indispensable for the survival of the secreting organism when encountered with an antagonistic organism in its surrounding.

## Author Contributions

All authors listed, have made substantial, direct and intellectual contribution to the work, and approved it for publication.

## Conflict of Interest Statement

The authors declare that the research was conducted in the absence of any commercial or financial relationships that could be construed as a potential conflict of interest.
